# Nociception and pain: lessons from optogenetics

**DOI:** 10.3389/fnbeh.2014.00069

**Published:** 2014-03-25

**Authors:** Fiona B. Carr, Venetia Zachariou

**Affiliations:** Fishberg Department of Neuroscience, Department of Pharmacology and Systems Therapeutics and Friedman Brain Institute, Icahn School of Medicine at Mount SinaiNew York, NY, USA

**Keywords:** pain, nociception, nociceptor, spinal cord, amygdala, prefrontal cortex

## Abstract

The process of pain perception begins in the periphery by activation of nociceptors. From here nociceptive signals are conveyed via the dorsal horn of the spinal cord to multiple brain regions, where pain is perceived. Despite great progress in pain research in recent years, many questions remain regarding nociceptive circuitry and behavior, in both acute nociception and chronic pain states. Techniques that allow for selective activation of neuronal subpopulations *in vivo* can provide a better understanding of these complex pathways. Here we review the studies to date that have employed novel optogenetic tools to improve our understanding of the pain pathway at the peripheral, spinal and supraspinal levels.

## Circuits and cell types involved in pain

Chronic pain represents a significant clinical problem affecting up to 20% of the general population (Breivik et al., 2006; Reid et al., 2011) and is a symptom of a variety of different underlying pathologies including arthritis, nerve injury, depression and cancer. An understanding of nociceptive mechanisms and the neurobiology of pain perception are necessary to better treat chronic pain conditions. Here we provide a brief overview of the circuitry and cell types involved, and review studies to date that have used optogenetic tools to manipulate these pathways.

Unlike some neurobiological disorders which can be attributed to specific brain regions, pain incorporates multiple components of the nervous system, at the peripheral, spinal, and supraspinal levels. The first components of the pain pathway are the peripheral nociceptors. These are a heterogeneous population, and can be classified in a variety of ways including myelination properties, expression of transducer molecules, peptidergic content and voltage gated sodium channel expression (Gold and Gebhart, 2010). Transgenic mice have been invaluable in demonstrating that nociceptor heterogeneity is of functional significance, with different nociceptors contributing to distinct nociceptive modalities such as heat and mechanical pain (Abrahamsen et al., 2008; Cavanaugh et al., 2009). Nociceptor subtypes also contribute in distinct ways to different forms of chronic pain such as inflammatory and neuropathic states (Cavanaugh et al., 2009; Seal et al., 2009; Minett et al., 2012). Activation of specific nociceptor subtypes in the intact system will be helpful in confirming modality specificity in pain, and determining appropriate peripheral targets for novel therapeutic strategies.

The dorsal horn of the spinal cord, where the majority of nociceptors terminate, provides the next level of complexity in pain processing. An exception to this are the trigeminal nociceptors, which innervate the head and facial areas and terminate in the medullary dorsal horn (Ren and Dubner, 1999). Interneurons are the most numerous population in the dorsal horn (Todd, 2010) and the balance between excitation and inhibition within the dorsal horn may be disrupted in chronic pain states. For instance, it is known that decreased GABAergic and glycinergic inhibition contribute to neuropathic pain hypersensitivity (Moore et al., 2002; Lu et al., 2013). In addition subtypes of glutamatergic interneurons may play distinct roles in acute, inflammatory and neuropathic pain states (Wang et al., 2013). To date our understanding of interneuron circuitry in the dorsal horn is based largely on electrophysiological and pharmacological approaches. Given the pivotal role of interneurons in the gate control theory of pain (Melzack, 1999), regional and cell type specific activation using optogenetics will be a powerful way to investigate this important circuitry.

**Figure 1:**
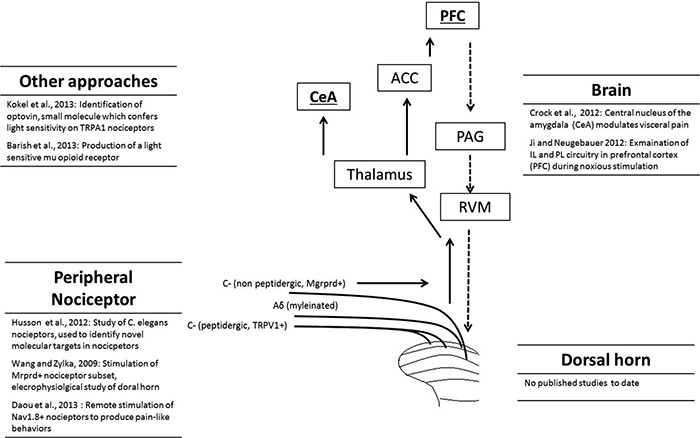
**Overview of the pain pathway, and list of optogenetic studies to date that have targeted each level**. The full arrows indicate the ascending pain pathway, while the descending pathway is represented by dashed arrows. Different categories of nociceptor, including unmyelinated C-fibers and myelinated Aδ-fibers are shown. For clarity, only a selection of brain regions activated during nociception are shown: prefrontal cortex (PFC), anterior cingulate cortex (ACC), central nucleus of the amygdala (CeA), parabrachial area (PB), periaqueductal gray (PAG) and rostral ventromedial medulla (RVM).

The principal direct targets of dorsal horn projection neurons are the parabrachial area and thalamus (Gauriau and Bernard, 2002, 2004). From here a range of areas are activated including the somatosensory cortex, anterior cingulate, prefrontal cortex (PFC), and amygdala (Apkarian et al., 2005; Tracey and Mantyh, 2007; Baliki et al., 2013). It is important to note that chronic pain states are frequently accompanied by psychological symptoms such as anxiety and depression (Bushnell et al., 2013) and understanding the role of brain areas in both the sensory and affective components of pain is important for the development of novel treatment approaches. Another important aspect of pain circuitry to be considered is the top-down pain modulatory system, which allows higher brain structures to signal to the dorsal horn to regulate nociceptive processing. This may be either inhibitory or facilitatory in nature, arises from a variety of brain structures and is relayed to the dorsal horn via the rostral ventromedial medulla (RVM; Ossipov et al., 2010). Optogenetic approaches will allow further investigation of these ascending and descending circuits in animal pain behavior (Figure 1).

### Optogenetic stimulation of nociceptors in C. elegans

C. elegans was the first multicellular organism to be investigated using optogenetic methods (Nagel et al., 2005), and is one of the most useful simple organisms in the study of genetics and molecular biology. Many advances in neuroscience and behavior have also been made using this system. C. elegans is particularly useful in the study of nociception as it exhibits a clear and reproducible withdrawal behavior, involving a reversal and change in direction away from the noxious stimulus (Wittenburg and Baumeister, 1999; Tobin and Bargmann, 2004). Recently, optogenetic activation of C. elegans nociceptors has been used to characterize molecules associated with nociception (Husson et al., 2012). Channelrhodopsin 2 was expressed under the control of the F49H12.4 promoter, predominantly found within PVD nociceptive neurons. Stimulation of the worm with blue light therefore allowed activation of the nociceptive network, without activating other non-noxious mechanical sensing neurons. Interestingly, activation of these nociceptors is less susceptible to habituation than other sensory responses in C. elegans. The authors used this technique to screen for genes involved in mediating the nociceptive response in the PVD neuron, investigating the contribution of RNA interference for various target genes to the optogenetically induced nociceptive behavior. This led to the identification of a number of genes involved in nociceptive transmission in the organism, including voltage gated calcium channel subunits and melastatin-related transient receptor (TRPM) channels. This study highlights the potential of using optogenetically triggered behavior as a method to screen for novel nociceptive mediators, or even analgesics.

### Optogenetic stimulation of specific nociceptor populations in mice

Optogenetic stimulation of a subset of peripheral nociceptor has also been used as a tool to characterize the spinal cord circuitry associated with that nociceptor subtype (Wang and Zylka, 2009). Mas-related G protein coupled receptor D (Mrgprd) is a marker for a subset of non-peptidergic nociceptors in the rat and mouse. These neurons appear to be involved in mediating mechanical nociception, but not noxious thermal or cold stimuli (Cavanaugh et al., 2009). Although anatomically it is known that Mrgprd-positive fibers terminate largely in lamina II of the dorsal horn, the precise connectivity of this nociceptor subpopulation was not known. In contrast to the variety of molecular markers available to characterize nociceptor populations, relatively few markers are available to characterize neuronal populations in the dorsal horn. To characterize the dorsal horn neurons innervated by Mrgprd expressing primary afferents, Wang and colleagues used a combined optogenetic and electrophysiological approach (Wang and Zylka, 2009). Knock-in mice were generated to express channlerhodopsin-2 at the Mrgprd locus, allowing this nociceptor population to be selectively activated by blue light. Patch clamp recordings from lamina II dorsal horn neurons were made using a slice preparation and stimulation of Mrgprd expressing terminals by brief high frequency blue light application. This was sufficient to induce excitatory post synaptic potentials in 50% of the lamina II neurons recorded. The non-responsive lamina II neurons are therefore likely to be innervated by other afferents, such as peptidergic nociceptors, supporting the view that pain processing is modality specific at both the peripheral and spinal levels. Interestingly this preparation illustrates that stimulation of channelrhodopsin-2 on axon terminals is sufficient to evoke post synaptic activity, as the dorsal root ganglions (DRGs) were not attached. This study highlights the potential of optogenetics in understanding the complex pain related circuitry within the dorsal horn. Unlike traditional electrophysiological studies which use electrical stimulation of afferents or application of a noxious stimulus, the optogenetic approach allows precise stimulation a defined nociceptor population. This is of great interest for the understanding connectivity between peripheral nociceptors and neurons in the spinal cord.

Recently, an alternative approach has been used to explore the use of optogenetics in pain studies. *Nav1.8* encodes a voltage gated sodium channel expressed in nociceptors, and Nav1.8 expressing primary afferent neurons have been previously shown to contribute to inflammatory and neuropathic pain (Abrahamsen et al., 2008). Many studies have used the Nav1.8-Cre mice as a background to study the role of particular genes in nociception and pain-like behaviors (Stirling et al., 2005). This mouse model has now been used to express channelrhodopsin-2 specifically in nociceptors. Stimulation of the hindpaw with blue light was sufficient to induce a number of characteristic nocifensive behaviors, including paw withdrawal, licking, jumping and vocalizations, in freely moving mice. Therefore this study provides the first evidence that optical stimulation of peripheral nociceptors can trigger nocifensive behaviors in the awake and freely moving animal (Daou et al., 2013). Furthermore, c-Fos labeling of the dorsal horn following this pattern of optical activation indicated activation of both lamina I and lamina II dorsal horn neurons, suggesting that both peptidergic nociceptors and non-peptidergic nociceptors are activated. Interestingly, the authors found that optogenetic stimulation of Nav1.8 nociceptors is also capable of driving long term hypersensitivity to mechanical and thermal stimuli, mimicking aspects of chronic pain models in mice. As pain has an important affective component, the authors also demonstrated that blue light could drive conditioned place aversion in these animals.

In contrast to the approach taken by Zlyka et al. in which channelrhodopsin was inserted into the locus of a low abundance marker of a subset of nociceptors, the Nav1.8-Cre approach may be more useful for behavioral studies, and investigations of spinal and supraspinal activation, given that a greater number of nociceptors will be activated in this way.

### Optogenetic studies of brain regions involved in pain

To date few studies have used optogenetic techniques to address the role of specific brain regions in pain behavior in rodents. One example has incorporated optogenetic stimulation to investigate the role of the central nucleus of the amygdala (CeA) in a mouse model of visceral pain (Crock et al., 2012). Visceral pain is a common complaint, and can be modeled in rodents by bladder or colorectal distension. In the study by Crock and colleagues, bladder distention was carried out, and the visceromotor response (VMR, elecromyographic response of the abdominal muscle) used as an index of visceral sensitivity. The amygdala is important in the affective dimensions of the pain response, and previous work had shown that the c-fos expression is increased in the CeA following noxious visceral stimulation (Han and Neugebauer, 2004). To explore this further, the right CeA was injected with herpes simplex virus (HSV) viral vector expressing channelrhodopsin-2. Optogenetic stimulation was carried out for 30 min before, during or after bladder distension. Optical stimulation before distension increased VMR sensitivity. This hypersensitivity was maintained for up to 15 min following the termination of the optical stimulation, providing direct evidence that neuronal activity in the CeA can lead to increased visceral nociception. A limitation of this study is the nature of the bladder distension protocol which requires the animals to be lightly anesthetized in order to perform measures of the VMR. Future studies to incorporate stimulation in awake, moving animals will provide further insights into the role of specific brain regions in the modulation of pain states.

Another important region involved in the modulation of pain is the PFC. As with the amygdala, this region is particularly associated with the affective component of the pain experience (Tracey and Mantyh, 2007). In a recent investigation, the PFC was injected with an adenoassociated virus (AAV), expressing channelrhodopsin-2 under the control of the calcium/calmodulin dependent kinase type II alpha (CaMKIIα) promoter (Ji and Neugebauer, 2012). This allows selective expression within pyramidal neurons only, which are the major excitatory population in the region, and avoids expression within inhibitory interneurons. Two distinct regions of the PFC were explored, the infralimbic (IL) and prelimbic (PL) areas. Following optogenetic stimulation of the IL region, extracellular recordings were made from pyramidal neurons in the IL and the nearby PL region with or without peripheral stimulation, and also following noxious mechanical stimulation of the knee joint. This approach helps provide a better understanding of circuitry in the PFC. Excitation of neurons in the IL region not only increases spontaneous activity of other neurons in this area, but also the evoked responses to noxious and non-noxious stimulation. Optical stimulation of the IL conversely led to a decrease in pyramidal neuron excitability in the PL, suggesting an inverse relationship between IL output and between PL activity (Ji and Neugebauer, 2012). This study is a good example of the type of analysis that can be achieved by combining optogenetic stimulation with electrophysiological recordings. Future studies may be useful to investigate the effects of chronic pain states, as opposed to acute noxious stimulation, on specific neuronal populations in the brain.

Recently we have applied an optogenetic approach to explore novel brain regions involved in opiate analgesia. Opioids are important clinically, however their use is limited through the development of tolerance and addiction (Ling et al., 2011). Understanding the brain regions and cell types involved in these processes are is important to help tackle the problem. We had previously demonstrated that regulator of G protein signaling 9-2 (Rgs9-2) is a negative modulator of morphine tolerance (Zachariou et al., 2003). In our recent study we demonstrated a potent role of Rgs9-2 in the nucleus accumbens (NAc, part of the brain reward center) in modulation of morphine tolerance. Taking advantage of an optogenetic strategy, we have showed that activation of Rgs9 expressing neurons in the NAc leads to the rapid development of analgesic tolerance in the hot plate test (Gaspari et al., 2014). Using bacterial artificial chromosome (BAC) lines expressing Cre recombinase, we were also able to selectively stimulate the two main NAc neuronal subpopulations: those expressing primarily D1 dopamine receptors (D1-type, direct pathway) and those enriched in D2 dopamine receptors (D2-type, indirect pathway). Activation of channelrhodopsin-2 in each of these neuronal subpopulation demonstrated that morphine tolerance is modulated by D1 type neurons, which is the population that mostly expresses mu opioid receptors (MORs; Cui et al., 2014). In accord with our earlier findings on a role of Rgs9-2 in the NAc in morphine tolerance, activation of D1-type but not D2-type neurons increases Rgs9-2 levels in the NAc (Gaspari et al., 2014).

### Optopharmacology and novel optoreceptors in pain research

Optogenetic approaches require the expression of an exogenous light sensitive molecule in the target system. Although this has proved a very useful tool in basic neurobiological research, by definition this approach is of limited use clinically. An alternative approach is the use of “optopharmacology”, that is administration of compounds that confer light sensitivity onto a specific cell type (Kramer et al., 2013). One recent example of this approach is the discovery of a novel small molecule, optovin, which confers light sensitivity on transient receptor potential cation channel, member A1 (TRPA1; Kokel et al., 2013). This ion channel is part of the transient receptor potential (TRP) family that act as transducers of noxious stimuli at the periphery. TRPA1 is activated by noxious cold and can also respond to chemical irritants such as formalin and mustard oil, and may also play a role in mediating mechanical nociception (Vay et al., 2012). The ability to optically stimulate peripheral fibers expressing TRPA1 is therefore of experimental interest to investigate the role of these neurons in animal and human pain sensation. Optovin was identified by screening a library of compounds in zebrafish embryos, which are normally insensitive to light. This molecule was found to confer sensitivity to violet light, and the effect was mediated by sensory neurons, as a spinalized preparation retained the response. A subset of mouse DRG cells also responded to optovin treatment, in a light dependent manner. These were identified as TRPA1 expressing nociceptors. Using zebrafish homozygous for TRPA1 it was demonstrated that this ion channel is required for the effects of optovin. Expression of human TRPA1 in cell line derived from human embryonic kidney (HEK) cells also conferred sensitivity to optovin.** The significance of this study lies in the ability to modulate the pain sensing TRPA1 neurons, without the need for genetic manipulations to confer this sensitivity. This approach may be useful for research and treatment of pain in the future. In particular, such an approach would allow selective optical control of subpopulations of peripheral nociceptors without the need for genetic manipulations.

Another approach has been the recent development of a photoactive MOR, the main receptor mediating the analgesic actions of morphine (Barish et al., 2013). This study designed a recombinant receptor, containing the optically active component of the rhodopsin molecule and the parts of the mu opioid receptor responsible for MOR receptor protein and G protein signaling. In a HEK cell system, this receptor is activated by light and has the ability to activate endogenous MOR receptor intracellular signaling pathways. Further development would be required, but expression of this mutant MOR receptor could be a useful way to overcome the side effects of MOR mediated analgesia, including analgesic tolerance, which is associated with alterations in MOR signal transduction pathways. Future work will determine if selective activation of the MOR in the periphery by light application prevents the CNS problems in patients requiring long term morphine treatment.

### Future directions in pain optogenetics

The power of optogenetics lies in the ability to achieve regional and cell type specific neuronal activation. These methods provide an unprecedented opportunity to probe the complexities of the pain pathway at the peripheral, spinal and supraspinal levels. Among the most exciting developments in the field to date is the ability to produce pain-like behaviors in transgenic mice by optical stimulation of nociceptors in the skin (Daou et al., 2013). This allows direct investigation of nociceptor activation in awake, freely moving animals, avoiding the stimulation of non-nociceptive neurons and other cell types in the periphery. The stimulus is also under precise temporal control, and avoids the need for using other artificial stimuli such as chemical irritants. Future studies to target specific subtypes of nociceptor will lead to greater advances in our understanding of modality specificity, and contribution of nociceptor subtypes to different forms of chronic pain.

Somewhat surprisingly, to date no studies have taken advantage of these tools within the dorsal horn however this may reflect technical challenges in inserting optogenetic fibers into the spinal cord. Considering the importance of this component of the pain pathway, however, it is likely that optogenetics will also provide valuable new insights into this complex circuitry. Interneuron based optogenetic experiments have been performed within the ventral horn of the spinal cord, to investigate the contribution of particular subsets to locomotor activity (Dougherty et al., 2013). It is feasible that a similar approach could be employed in the dorsal horn, given our increasing anatomical and molecular knowledge of interneuron populations (Todd, 2010).

As described, a small number of studies have explored brain areas in pain using optogenetics, however many other areas of the pain matrix remain to be studied in this way. Both brain studies to date have relied either on electrophysiological recordings (Ji and Neugebauer, 2012) or behavioral outcomes in the anesthetized animal (Crock et al., 2012). One of the most important directions to be explored will be the combination of optogenetic brain stimulation with behavior in the awake animal.

The studies described here are only the beginning of what we expect to be fruitful and informative exploration of pain circuits using optogenetic tools. It is likely that subsequent studies will move beyond these initial proof of concept studies, and use optogenetic tools to tackle unanswered questions regarding pain circuitry.

## Conflict of interest statement

The authors declare that the research was conducted in the absence of any commercial or financial relationships that could be construed as a potential conflict of interest.
